# Biochar enhances cucumber production by modulating rhizosphere microbiota and soil metabolites under continuous cropping systems

**DOI:** 10.3389/fpls.2026.1726191

**Published:** 2026-05-21

**Authors:** Guoping Xue, Yun Hu, Haifeng Xue, Xueyu Wang, Hongmei Bai, Jinwei Du, Yaping Wang, Hongli Huo, Ming Li, Wei Jiang

**Affiliations:** 1 College of Horticulture and Plant Protection, Inner Mongolia Agricultural University, Hohhot, China; 2 Inner Mongolia Academy of Agricultural and Animal Husbandry Sciences, Hohhot, China; 3 Vocational and Technical College of Inner Mongolia Agricultural University, Baotou, China

**Keywords:** bacterial community, biochar, cucumber, metagenomic analysis, untargeted metabolomics

## Abstract

Biochar, a soil amendment with diverse regulatory functions, has been widely applied to enhance soil conditions. However, its underlying mechanism for alleviating continuous cropping obstacles, from the perspective of rhizosphere microbe-metabolite-plant coupling, remains to be further elucidated. Using cucumber (*Cucumis sativus* L.) as the model crop, this study explored the rhizosphere-mediated effects of biochar application under continuous cropping conditions via the analytical methods of metagenomics and metabolomics. Six biochar application rates (0, 5, 10, 20, 30, and 40 t ha^-^¹) were tested. All biochar treatments significantly improved cucumber yield by 20%-50%, with the C30 and C40 treatments producing the most pronounced yield enhancement. C10, C20, C30 and C40 treatments had a positive effect on cucumber quality, soil physicochemical properties and enzymatic activities. Vitamin C and soluble protein peaked in C20, whereas some sugar indicators decreased across all biochar treatments. Urease activity was significantly elevated under C20, C30, and C40 treatments. Notably, the C40 treatment led to marked increases in total nitrogen, available phosphorus, and sucrase activity. Biochar amendments also enriched key bacterial phyla involved in carbon and nitrogen cycling, including Actinobacteria, Bacteroidetes, Chloroflexi, and Bacillota. Medium to high application rates (C20, C30, C40) upregulated various secondary metabolic pathways associated with biotic stress resistance, including the biosynthesis pathways of phenylpropanoids, various alkaloids, and the metabolic pathway of phenylalanine. High biochar application rate (C40) characterized lipid metabolism as the core responsive pathway and significantly downregulated galactose metabolism. This study reveals that biochar application represents a promising strategy to mitigate continuous cropping obstacles of cucumber by enhancing nutrient cycling, enzyme activities, soil metabolite composition, and the rhizosphere microbial community in facility systems of the cold and arid northern regions of China.

## 1 Introduction

Cucumber (*Cucumis sativus* L.) represents a major vegetable crop cultivated extensively in China. It is highly valued by both consumers and producers due to its high yield, favorable economic returns, and rich nutritional profile (Wang et al., 2024). Protected cultivation has become the dominant agricultural practice in Northern China, with cucumber emerging as one of the region’s primary cash crops. However, the high production intensity, elevated multiple cropping index, prolonged reliance on chemical fertilizers, and simplified cropping systems typical of protected agriculture have collectively intensified the challenges associated with continuous cropping (Zhao et al., 2025). Continuous monoculture leads to the selective uptake of ions by plant roots, resulting in the accumulation or depletion of specific ions. This imbalance ultimately contributes to secondary soil salinization and nutrient disorder. Continuous cropping also reduces soil organic matter while promoting the accumulation of nitrate nitrogen and available phosphorus in soils under protected cultivation (Liu et al., 2025). Zhen et al. (2021) demonstrated that nutrient imbalances induced by continuous cropping are a principal factor disrupting the coordinated uptake of phosphorus, nitrogen, and potassium in cucumber leaves, with detrimental consequences for plant growth and yield. Long-term continuous cropping further suppresses soil enzyme activities, thereby limiting the efficiency of nutrient utilization (Jin et al., 2023; Tong et al., 2023; Zhao et al., 2023; Zhao et al., 2025). Numerous studies suggested that continuous cropping significantly alters the composition and structure of soil microbial communities, resulting in a decline in microbial diversity. These shifts negatively impact plant development, nutrient cycling, and overall ecosystem functionality (Ma et al., 2023; Li et al., 2025; Shen et al., 2025; Wang et al., 2025). Moreover, continuous cropping modifies the profile of root-secreted secondary metabolites. The resulting allelopathic substances exert stronger inhibitory than stimulatory effects. This shift is accompanied by a marked decline in soil carbon-nitrogen metabolites—such as organic acids, carbohydrates, and amino acids—along with downregulation of microbial functional genes related to carbon and nitrogen metabolism. Consequently, carbon-nitrogen cycling efficiency is reduced, with urease and invertase activities declining by 30%~50%, ultimately decreasing nutrient use efficiency (Chen et al., 2022; Zheng et al., 2022; Ding et al., 2024). Additionally, sustained release of compounds such as aldehydes, ketones, phenols, and organic acids disrupts nutrient uptake and interferes with various physiological and biochemical processes, impairing root vitality and further inhibiting the growth of cucumber shoots and roots (Li et al., 2024).Therefore, the development of eco-friendly and effective strategies to mitigate the adverse effects of continuous cropping is imperative. Such efforts are crucial to ensuring the sustainable and healthy progression of China’s facility vegetable industry.

Research on the use of biochar as a soil amendment to mitigate continuous cropping obstacles is extensive within agricultural systems (Zhou et al., 2024; Ding et al., 2025). Biochar has been shown to increase soil organic matter and the availability of essential nutrients such as nitrogen, phosphorus, and potassium (Huang et al., 2023; Zhang et al., 2024), enhance soil enzyme activity (Huang et al., 2025), and demonstrate significant potential to improve soil productivity (Liu et al., 2022). Its yield-promoting effects have been validated across a variety of crop types, including the three major staple crops, Chinese cabbage, and cucumber (Wang et al., 2021; Fan et al., 2024; Zhu et al., 2025). As key decomposers, soil microorganisms play a crucial role in material cycling and energy flow within terrestrial ecosystems, serving as critical determinants of plant health and productivity (Wang et al., 2023). Biochar has been found to modify microbial community composition, promote nutrient accumulation, and ultimately increase maize yield (Han et al., 2023). Additionally, biochar alters the abundance of nitrogen metabolism-related microbial groups such as Proteobacteria, Cyanobacteria, and Sphingomonas, thereby enhancing *plants*’ nitrogen uptake capacity and promoting cucumber growth (Yang et al., 2024). Appropriate biochar application increases soil bacterial diversity and fosters the proliferation of beneficial microorganisms that antagonize pathogens, resulting in increased pepper yield and effective alleviation of continuous cropping obstacles in pepper cultivation (Xu et al., 2025). Furthermore, biochar-based organic fertilizer promotes the enrichment of beneficial bacterial genera such as *Pseudomonas* and *Gemmatimonas*, suppresses the abundance of pathogenic microorganisms, including Alternaria and Fusarium oxysporum, decreases disease incidence in tobacco, and enhances tobacco leaf yield (Chen et al., 2025). Soil metabolites, derived from plant roots and microorganisms, reflect both direct and historical responses of soil microbial communities to soil nutrient status. Root exudates facilitate the accumulation of soil available nutrients (Wang et al., 2025), while rhizodeposition of organic molecules increases nutrient availability and provides sufficient substrates to support microbial growth in the rhizosphere (Lu et al., 2023; Von Haden et al., 2024). Furthermore, microbial metabolism of such organic compounds in rhizosphere soil may impact soil microbial community composition and root physiological activities (Gong et al., 2023).

Soil enzyme activity, organic matter content, nutrient availability, plant nutrient uptake, productivity, microbial biomass, metabolite profiles, and other relevant indices are commonly employed to assess ecosystem functions and services (Tang et al., 2022; Xu et al., 2023). Consequently, a comprehensive analysis of soil rhizosphere bacterial community assembly alongside changes in soil metabolites represents an effective approach for evaluating soil productivity. However, comprehensive studies investigating how biochar application influences the integrated interactions among plant performance, soil physicochemical properties, microbial community composition, and rhizosphere metabolite dynamics in continuous cucumber cropping systems remain limited. In this study, soil samples were collected from continuous cucumber cropping fields in the cold, arid regions of northern China. We evaluated the effects of gradient biochar application rates on rhizosphere bacterial diversity, metabolite profiles, and cucumber yield. The primary objective was to elucidate the synergistic regulatory mechanisms within the soil-microbe-metabolite-plant continuum under biochar amendment, identify the regionally optimal biochar application rate, and provide novel theoretical support for the sustainable mitigation of continuous cropping obstacles in protected vegetable cultivation systems.

## 2 Materials and methods

### 2.1 Trial site and setup

A short-term field experiment was conducted from spring to autumn in 2023 in Taobuqi Village, Yulin Town, Saihan District, Hohhot, Inner Mongolia, China (41°54′N, 122°6′E). The soil at the site was classified as black loessial soil, predominantly exhibiting a sandy loam texture. Prior to the experiment, the 0–20 cm soil layer demonstrated the following characteristics: pH 7.10; organic matter content of 82.94 g kg^-1^; total nitrogen (TN) of 4.64 mg g^-1^; total phosphorus (TP) of 2.44 g kg^-1^; available nitrogen (AN) of 264.59 mg kg^-1^; available phosphorus (AP) of 120.04 mg kg^-1^. The cucumber variety utilized in this study was ‘Bomei’ (Supplied by Tianjin Deruite Seed Industry Co., Ltd.). This cultivar exhibits vigorous growth and continuous fruiting performance, with a low malformation rate of fruits. It also demonstrates strong disease resistance and is highly adaptable to protected cultivation systems, such as greenhouse cultivation, in northern China.

The biochar applied in the experiment was produced by pyrolyzing corn stover under oxygen-limited conditions at temperatures ranging from 350-550 °C (Hongyuan Jialian Biomass Energy Development Company, Jilin Province, China), and it underwent a 60-day natural aging process prior to field application. The fundamental physical and chemical properties of the biochar are summarized in **Supplementary Table 1**.

In this field study, six treatments were applied: no biochar application (CK) and biochar application rates of 5 (C5), 10 (C10), 20 (C20), 30 (C30), and 40 t ha^-1^ (C40). Each treatment was replicated in three plots measuring 1.6 m × 6.5 m, arranged in a randomized block design. During the experimental period, cucumber seedlings were transplanted on January 17, 2023, with a row spacing of 30 cm and a plant spacing of 17 cm. Biochar was manually applied once in early spring to the designated plots and incorporated into the soil. Film-covered drip irrigation was used uniformly across all plots, and other field management practices were consistent throughout the experiment.

### 2.2 Soil sampling collection

Following the cucumber harvest in autumn 2023, soil samples from the 0~20 cm soil layer were collected using the shaking root method (Caporaso et al., 2010). For each treatment, five samples were obtained from randomized locations, with three replicates. The collected soil samples were homogenized and divided into two equal portions. One portion was placed in sealed, virus-free bags, transported on dry ice immediately, and stored at -80 °C for subsequent analysis of rhizosphere soil metabolites. The other portion was air-dried naturally, sieved through a 2 mm mesh, and used for the determination of soil physicochemical properties and soil enzyme activities.

### 2.3 Cucumber yield

Fruits were harvested in multiple batches at different maturity stages. The number of fruits per plant and the single fruit weight were recorded, followed by calculations of fruit yield per plant, plot yield, and total yield.

### 2.4 Cucumber quality

The contents of vitamin C (VC), soluble protein (SP), soluble sugar (SS), reducing sugar (RS), and total sugar (TS) were determined as follows: VC by the Soluble Blue Salt B colorimetric method, SP by the Coomassie Brilliant Blue method, SS by the anthrone colorimetric method, and RS and TS by the 3,5-dinitrosalicylic acid (DNS) colorimetric method.

### 2.5 Soil physicochemical properties

Soil indices were analyzed according to Soil Agrochemical Analysis (Bao, 2000). TN and total carbon (TC) were determined using a German Elemental Analyzer (Vario MACRO cube). TP and AP were measured using the molybdenum-antimony anti-colorimetric method. AN was determined by the alkaline hydrolysis diffusion method.

### 2.6 Enzyme activity

Soil urease (URE) activity was determined using the indophenol blue method (Luo et al., 2025). The activities of soil sucrase (SUC), and alkaline phosphatase (ALP) were measured using biochemical assay kits.

### 2.7 DNA extraction and metagenomic sequencing

DNA was extracted using the E.Z.N.A.^®^ soil DNA kit (Omega Bio-tek, Norcross, GA, U.S.). The concentration and purity of extracted DNA was determined with SynergyHTX and NanoDrop2000, respectively. DNA quality was checked on 1% agarose gel. The extracted DNA was fragmented to an average size of approximately 350 bp using a Covaris M220 instrument (Gene Company Limited, China) for paired-end library construction. The paired-end library was prepared using the NEXTFLEX Rapid DNA-Seq kit (Bioo Scientific, Austin, TX, USA). Paired-end sequencing was conducted on an Illumina NovaSeq™ X Plus platform (Illumina Inc., San Diego, CA, USA) at Majorbio Bio-Pharm Technology Co., Ltd. (Shanghai, China). The raw sequencing data have been deposited in the NCBI database under accession number PRJNA1342099 (http://www.ncbi.nlm.nih.gov/bioproject/1342099).

### 2.8 Rhizosphere soil metabolites extraction and determination

100 mg solid sample was added to a 2 mL centrifuge tube, and a 6 mm diameter grinding bead was added. 1000 μL of extraction solution (methanol: water = 4:1,v/v) containing four internal standards (0.02 mg/mL, L-2-chlorophenylalanine, etc.) was used for metabolite extraction. Samples were frozen in a tissue grinder for 6 min (-10 °C, 50 Hz), followed by low-temperature ultrasonic extraction for 30 min (5 °C, 40 kHz). The samples were incubated at -20 °C for 30 min, centrifuged for 15 min (4 °C, 13000 × g), and the supernatant was transferred to the injection and analyzed using an ultra-high-performance liquid chromatography-orbitrap high-resolution mass spectrometer at Majorbio Bio-Pharm Technology Co., Ltd. (Shanghai, China).

After the instrumental analysis was completed, the LC-MS raw data were imported into the metabolomics data processing software Progenesis QI(Waters Corporation, Milford, USA) for baseline filtering, peak detection, integration, retention time correction and peak alignment. A data matrix containing retention time, mass-to-charge ratio, and peak intensity was ultimately generated. Subsequently, the software was used for characteristic peak database matching and metabolite annotation. Meanwhile, the MS and MS/MS spectral data were matched against the public metabolomics databases, including the Human Metabolome Database (HMDB, https://hmdb.ca/), Metlin (https://metlin.scripps.edu/). The data matrix generated after database searching was uploaded to the Majorbio Cloud Platform (https://cloud.majorbio.com/) for further analysis.

### 2.9 Data processing and statistical analysis

Cucumber yield, Cucumber quality, soil physicochemical properties, and enzyme activities are presented as mean values ± SEM. Statistically significant differences between soil groups were obtained using one-way ANOVA (*p* < 0.05; Tukey HSD test) with the SPSS software (version 2024, IBM). Microorganism and metabolomics data analyses were performed on the Meiji Cloud platform (https://v.majorbio.com/project-center/overview).

## 3 Results

### 3.1 Biochar application rates altered the yield and quality of cucumber

**Figure 1:**
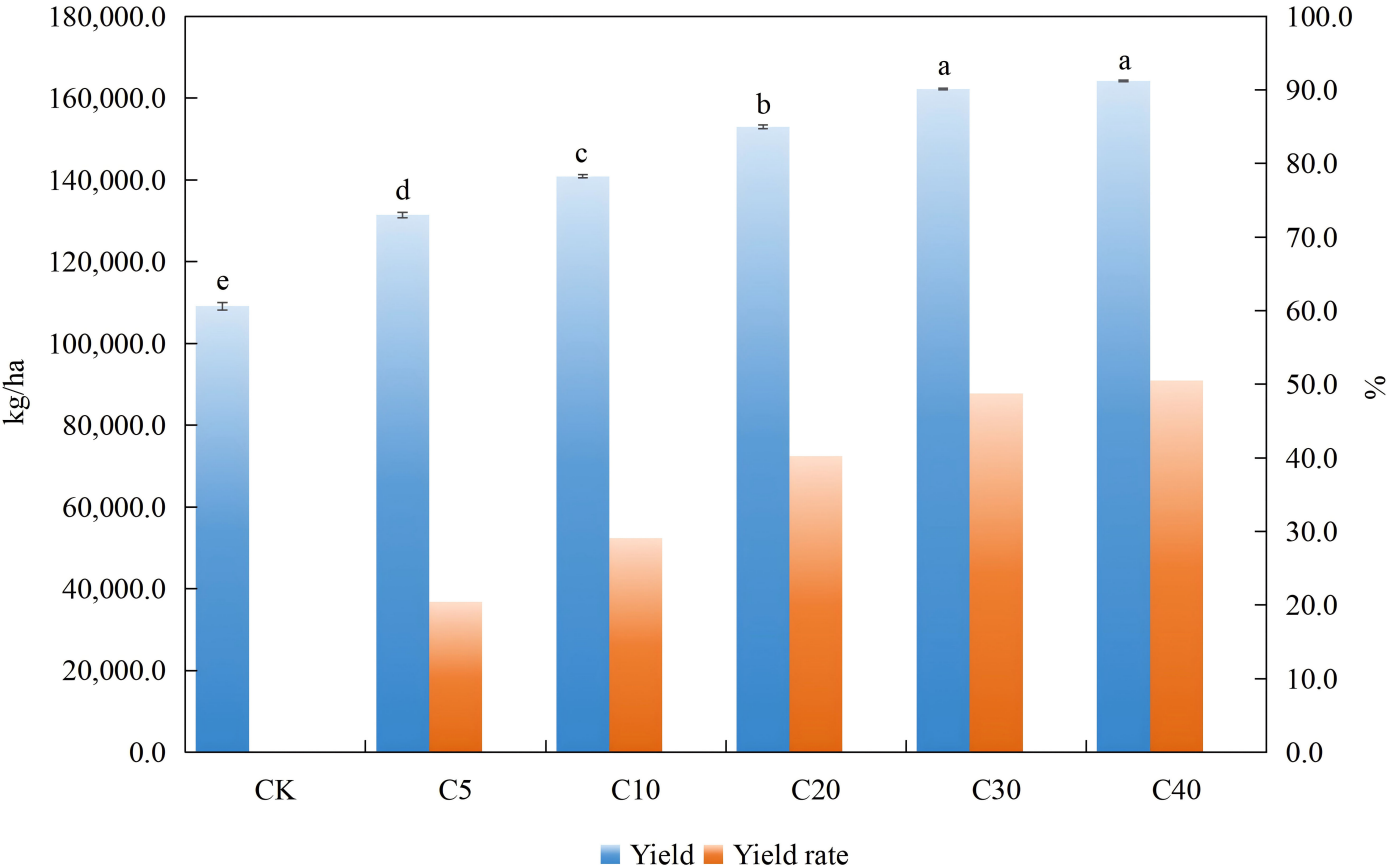
Effect of biochar application rate on cucumber yield and yield increase rate. Different lowercase letters within the same column indicate statistically significant differences among treatments at p < 0.05.

Different application rates of biochar significantly improved cucumber yield. The cucumber yields under various biochar treatments are illustrated in **Figure 1**. Compared with the control (CK), the yields for treatments C5, C10, C20, C30, and C40 increased by 20.45%, 29.10%, 40.25%, 48.73%, and 50.51%, respectively. Different application rates of biochar regulated cucumber quality to some extent under continuous cropping conditions, with several indices reaching a significant level (**Table 1**). Compared with CK, the vitamin C content exhibited an increasing trend with higher biochar application rates, a significant elevation was observed in the C20 treatment. Soluble protein content increased across all treatments, with significant elevation observed in C10, C20, C30 and C40 treatments, peaking in the C20 treatment. The total sugar content was significantly increased only in the C5 treatment, whereas it was decreased in other treatments. Notably, the reductions in C20 and C30 treatments were statistically significant. Soluble sugar and reducing sugar contents decreased across all biochar treatments. Specifically, soluble sugar content was significantly reduced in the C40 treatment, whereas significant declines in reducing sugar content were observed in C5, C10, C20 and C40 treatments.

**Table 1:** Effect of biochar application on cucumber quality.

Treatment	VC (μg g^-1^)	TS (mg g^-1^)	SS (mg g^-1^)	RS (mg g^-1^)	SP (mg g^-1^)
CK	103.86 ± 5.38b	43.20 ± 0.86b	26.01 ± 1.36a	22.79 ± 0.70a	15.66 ± 0.54c
C5	105.46 ± 6.31ab	47.41 ± 1.26a	24.38 ± 0.85ab	19.49 ± 0.87b	15.54 ± 0.74c
C10	105.31 ± 8.30ab	40.27 ± 0.64bc	24.89 ± 1.29ab	19.00 ± 1.19b	21.53 ± 1.75b
C20	119.73 ± 4.23a	38.93 ± 1.61c	24.58 ± 1.20ab	18.60 ± 1.32b	32.58 ± 1.04a
C30	117.43 ± 4.43ab	42.32 ± 1.48b	24.49 ± 0.33ab	22.49 ± 1.10a	21.94 ± 1.14b
C40	114.20 ± 5.06ab	41.61 ± 0.20bc	22.07 ± 0.98b	19.62 ± 0.09b	21.30 ± 0.03b

Different lowercase letters within the same column indicate statistically significant differences among treatments at *p* < 0.05.

### 3.2 Effects of biochar additions on soil enzyme activities and physicochemical properties

To assess whether enzyme activities varied in response to changes in the functional bacteria within biochar-amended rhizosphere soils, the activities of soil SUC, URE, and ALP were measured (**Table 2**). The results demonstrated that biochar application at varying rates significantly enhanced soil URE activity. Compared with the CK, URE activities in C20, C30 and C40 treatments increased by 13.17%, 17.40%, and 31.22%, respectively, with the C40 treatment exhibiting significantly higher activity than both C20 and C30. Similarly, the C20, C30, and C40 URE activities in C20, C30 and C40 treatments increased, with the C40 treatment producing a significant increase of 37.66%. No significant difference was observed between the C30 and C40 treatments. However, the C40 treatment showed significantly higher SUC activity than the other biochar application treatments. Biochar application had minimal impact on soil ALP activity. Given that soil URE and SUC are integral to the nitrogen and carbon cycles, respectively, these results are significant. Soil URE catalyzes the hydrolysis of organic nitrogen, converting it into plant-available nitrogen (Dawar et al., 2021), while soil SUC hydrolyzes sucrose, thereby supplying carbon sources and energy to the soil ecosystem (Li et al., 2025). Consequently, the elevated activities of URE and SUC in biochar-amended soils may contribute to enhanced soil productivity.

**Table 2:** Effect of biochar application on soil physicochemical properties and enzyme activities in rhizosphere soil.

Treatment	TC(g/kg^-1^)	TN(g/kg^-1^)	TP(g/kg^-1^)	AN(mg/kg^-1^)	AP(mg/kg^-1^)	URE(mg/d^-1^/g^-1^)	SUC(mg/d^-1^/g^-1^)	ALP(mg/d^-1^/g^-1^)
CK	54.14 ± 4.50a	5.82 ± 0.47b	2.80 ± 0.12a	282.81 ± 16.82a	342.92 ± 14.88b	6.15 ± 0.21c	28.145 ± 2.50b	5.41 ± 0.22a
C5	54.36 ± 1.40a	5.76 ± 0.36b	2.64 ± 1.71a	278.22 ± 11.19a	342.50 ± 9.92b	6.71 ± 0.20bc	24.70 ± 1.17b	5.45 ± 0.10a
C10	58.70 ± 4.98a	5.76 ± 0.11b	2.73 ± 0.05a	273.87 ± 9.46a	356.67 ± 0.72b	6.7 ± 0.12bc	27.33 ± 1.39b	5.16 ± 0.39a
C20	58.11 ± 1.73a	6.30 ± 0.28ab	2.49 ± 0.14a	287.86 ± 5.85a	331.25 ± 12.05b	6.96 ± 0.29b	29.37 ± 4.53b	5.59 ± 0.19a
C30	55.41 ± 1.03a	6.54 ± 0.60ab	2.58 ± 0.17a	280.75 ± 6.12a	337.08 ± 9.21b	7.22 ± 0.26b	30.88 ± 3.86ab	5.44 ± 0.05a
C40	58.68 ± 1.50a	7.08 ± 0.22a	2.84 ± 0.14a	276.62 ± 3.15a	463.33 ± 5.05a	8.07 ± 0.39a	38.75 ± 3,68a	5.41 ± 0.24a

Different lowercase letters within the same column indicate statistically significant differences among treatments at *p* < 0.05.

The physicochemical properties of the soil were subsequently analyzed (**Table 2**). The results indicated that the addition of biochar exerted little effect on soil TC content, AN content, and TP content. However, the C40 treatment significantly increased the concentrations of TN and AP. As important indicators of soil nutrients, TN and AP serve as key measures of soil fertility and productivity (Jiang et al., 2025).

### 3.3 Biochar application rates reshaped rhizosphere microbiome

#### 3.3.1 Response of alpha diversity and beta diversity to biochar

**Figure 2:**
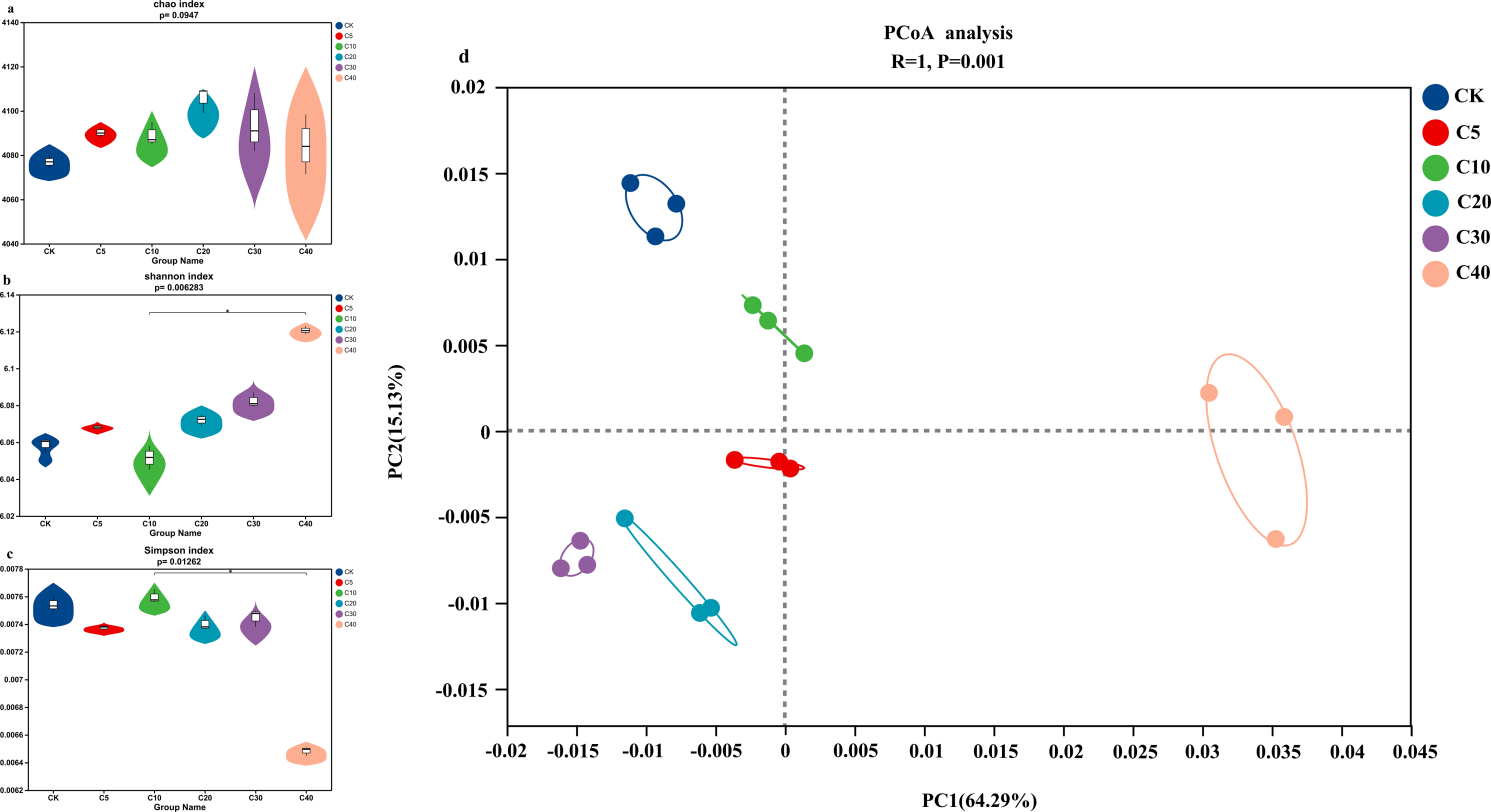
Alpha and beta diversity analyses of rhizosphere bacterial communities under different biochar treatments: **(a)** Chao1 richness index, **(b)** Shannon diversity index, and **(c)** Simpson diversity index showing alpha diversity metrics; **(d)** PCoA based on Bray-Curtis dissimilarity reveals clustering patterns among treatments. (Points of the same color represent replicates from the same treatment group, with ellipses indicating group dispersion). Asterisks indicate significance levels, *,** and *** indicate P < 0.05, P < 0.01 and P<0.001 respectively.

The research conducted alpha diversity analysis to assess the richness and diversity of bacterial communities across different treatment groups. The results indicated that biochar treatments at varying application rates had minimal impact on the richness index (Chao index) and diversity indices (Shannon index and Simpson index; **Figures 2a–c**). Additionally, beta diversity analysis was performed to investigate the similarities and differences among soil samples. Principal coordinate analysis (PCoA) revealed significant separations between biochar-treated groups and the CK, as well as among the different biochar treatments, suggesting that biochar application rates impacted the bacterial community structure in cucumber continuous cropping soil (**Figure 2d**). These findings were further supported by analysis of similarity (ANOSIM, R = 1, *p* < 0.001).

#### 3.3.2 Enriched differential microorganisms under different biochar treatments

**Figure 3:**
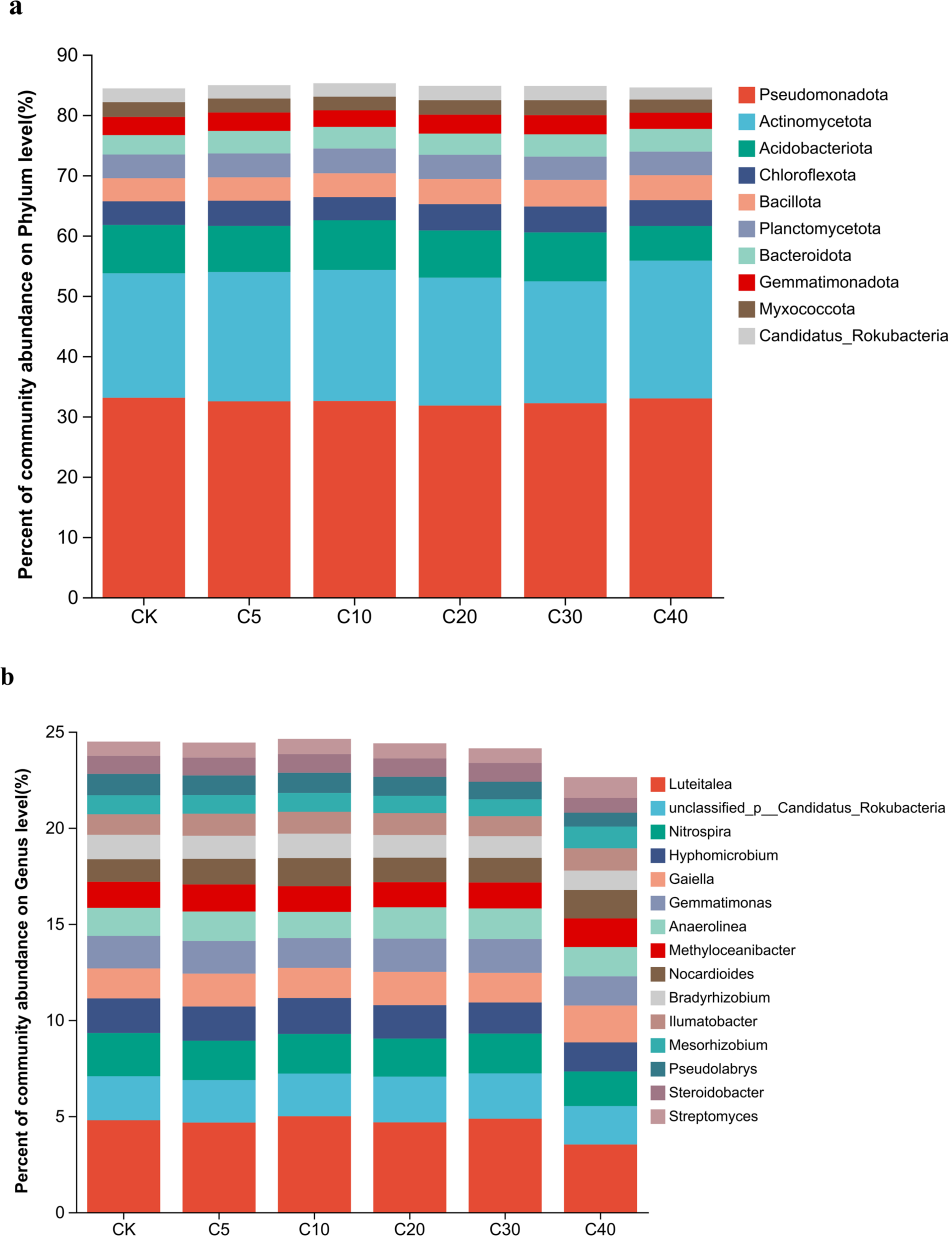
Composition and differential enrichment of rhizosphere bacterial communities under biochar treatments: **(a)** Relative abundance of bacterial taxa at phylum level **(b)** Relative abundance of bacterial taxa at genus level.

Proteobacteria, Actinobacteria, Acidobacteriota, Chloroflexota, Bacillota, Planctomycetota, Bacteroidota, and Gemmatimonadota were the dominant phyla across all samples, collectively accounting for approximately 80% of the total phyla (**Figure 3a**). At the genus level, the relative abundance of individual bacterial genera was below 5%, indicating a high diversity of the bacterial community at this taxonomic level (**Figure 3b**). The differential bacterial genera identified in soils predominantly affiliated with the dominant phyla. Differentially abundant genera in each treatment group were identified through linear discriminant analysis effect size (LEfSe) analysis, and linear discriminant analysis was employed to assess their differential effects (**Supplementary Figures 1a-e**). The results demonstrated different microbial enrichment patterns associated with varying biochar application rates. Proteobacteria, Acidobacteriota, Gemmatimonadota, and Myxococcota were enriched in the CK treatment. Biochar application at different rates significantly altered in the relative abundances of the key microbial taxa to varying degrees. C5, C10, C20, and C30 treatments significantly decreased the relative abundance of Proteobacteria, whereas the C40 treatment resulted in the most pronounced reduction in the relative abundances of Acidobacteriota, Gemmatimonadota, and Myxococcota (**Figure 3a**). Whereas Actinobacteria, Bacteroidetes, Bacillota and Chloroflexi were significantly enriched following biochar application. C10 and C40 treatments significantly increased the relative abundance of *Actinobacteriot*a; All biochar application rates notably elevated the relative abundance of Bacteroidota. The C30 treatment markedly enhanced the relative abundance of Bacillota, and C5, C20, C30, and C40 treatments significantly raised the relative abundance of Chloroflexota (**Figure 3a**). At the genus level, *Nitrospira*, *Bradyrhizobium*, *Pseudolabrys*, *Hyphomicrobium*, *Steroidobacter*, and *Gemmatimonas* were dominant in the CK treatment, with their abundances significantly reduced to varying degrees under different biochar treatments (**Supplementary Table 2**). *Luteitalea* was the dominant genus in the C10 treatment, while its abundance significantly decreased in the C40 treatment. *Nocardioides* was dominant in both C10 and C40 treatments, exhibiting a significant increase across all biochar treatments. *Gaiella* was dominant in C20 and C40 treatments, with its abundance significantly increased in C5, C20, and C40 treatments. *Streptomyces* was dominant in the C40 treatment, with its abundance significantly elevated in C10 and C40 treatments.

### 3.4 Response of soil metabolites to biochar in continuous cucumber soil

#### 3.4.1 PLS-DA analysis

**Figure 4:**
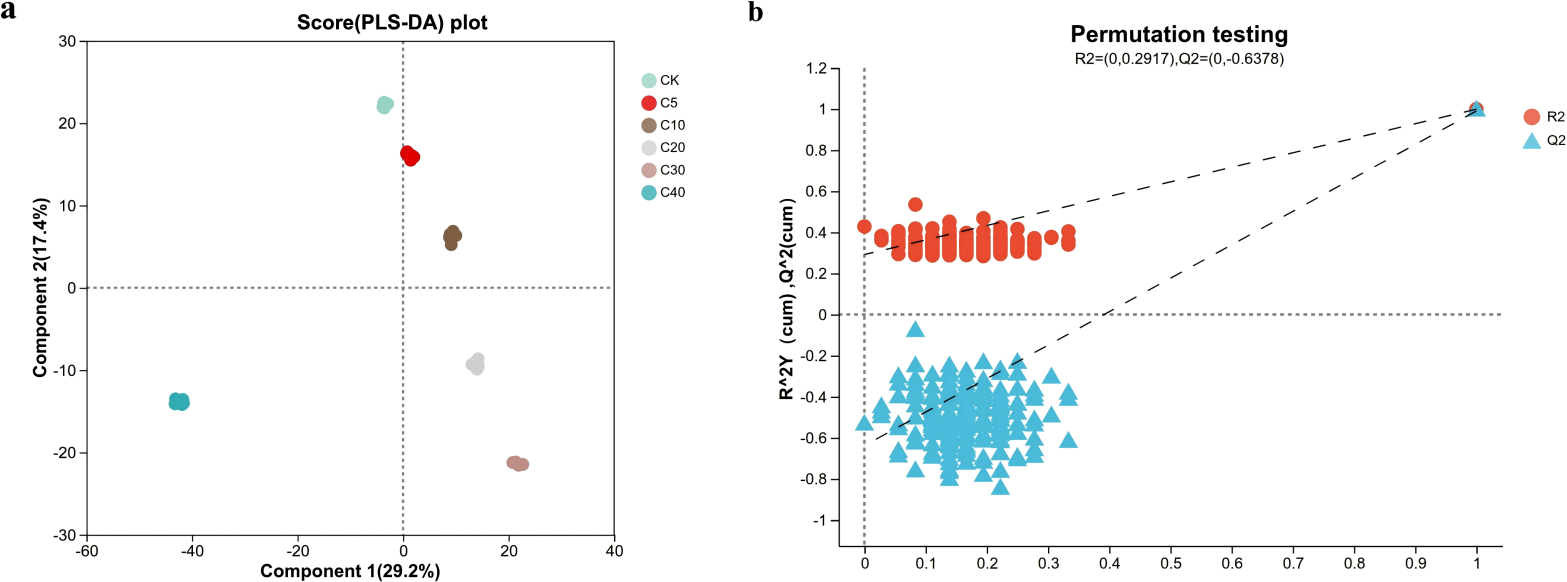
PLS-DA and model validation of rhizosphere soil metabolites: **(a)** PLS-DA score plots illustrating separation of treatment groups under mix ionization modes; **(b)** Permutation test results validating robustness of PLS-DA models for mix ion modes. (The x-axis represents the permutation retention rate, while the y-axis shows R^2^ values (blue dots) and Q^2^ values (red triangles); dashed lines indicate the regression trends of R^2^ and Q^2^, confirming no model overfitting).

Comparative analysis among the five groups suggested that different biochar application rates significantly altered the soil metabolite profiles. The partial least squares-discriminant analysis (PLS-DA) score plots (**Figure 4**) demonstrate the separation and classification among treatment groups. In **Figure 4a**, a clear separation between biochar treatments and CK was observed under mixed positive and negative ion mode. The distance between each treatment group and the CK increased progressively with higher biochar application rates, and distinct separation among treatments was also evident. These results suggest that biochar application differentially alters the rhizosphere soil metabolite composition of cucumber. Permutation tests (**Figure 4b**) confirmed the reliability of the PLS-DA model. As the permutation retention rate decreased, both R² and Q² values declined monotonically, while the intercept increased linearly, confirming model validity and the absence of overfitting.

#### 3.4.2 Metabolites analyzed by Venn diagram and volcano plots

**Figure 5:**
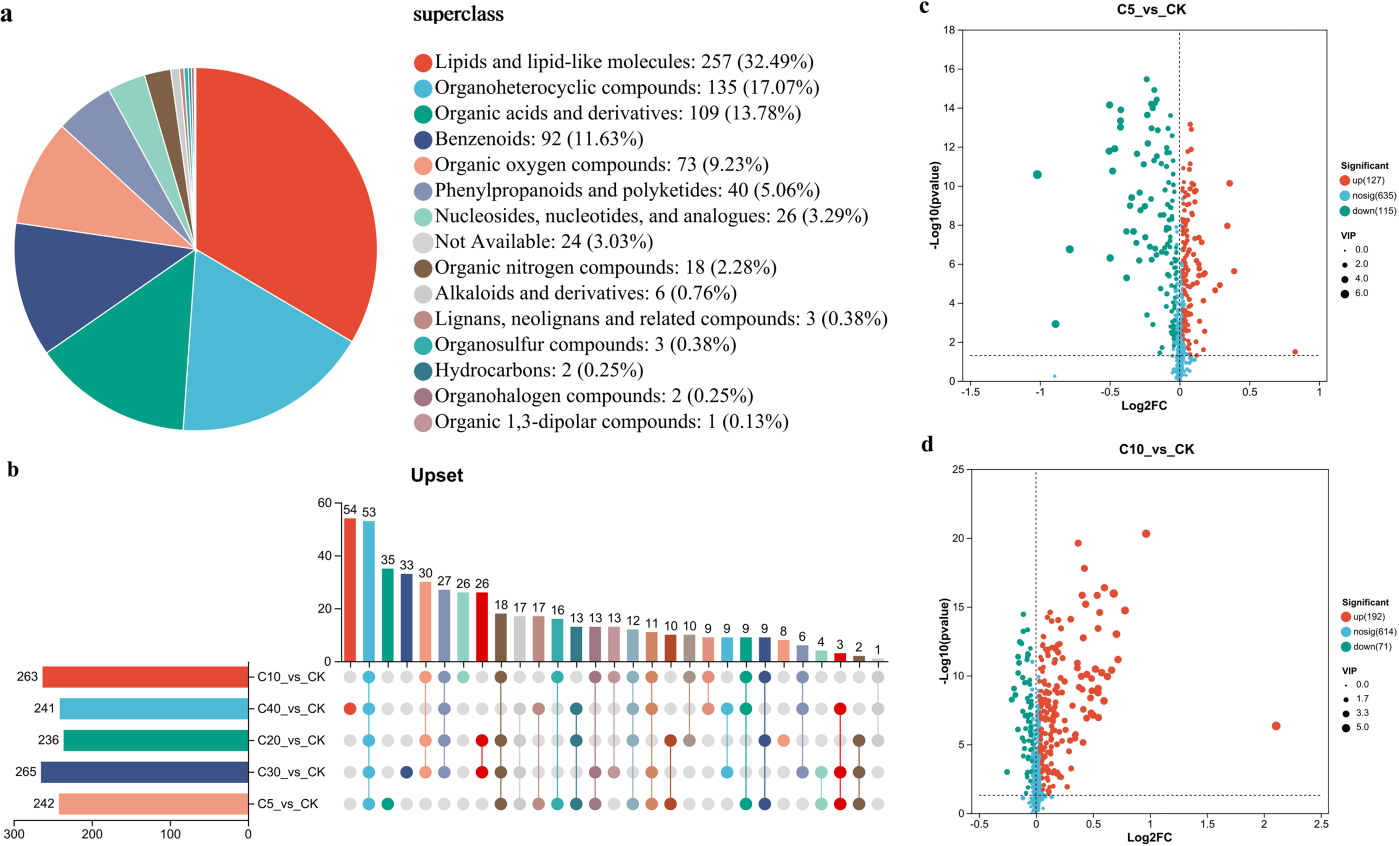
Taxonomic annotation and metabolite distribution in the rhizosphere across biochar treatments and CK: **(a–d)** HMDB-based taxonomic annotation of detected metabolites, classified into major compound categories **(a)** Upset diagram illustrating the number and overlap of DAMs across treatment comparisons with CK **(b)** Volcano plots showing the distribution of DAMs in C5 vs. CK **(c)** and C10 vs. CK **(d)**. (Each dot represents a unique metabolite, with size indicating the VIP (Variable Importance in Projection) score. Red dots indicate significantly upregulated metabolites, green dots represent significantly downregulated metabolites, and blue dots denote metabolites with no significant change (VIP > 1 and *p* < 0.05).).

To elucidate the taxonomic composition of rhizosphere metabolites under different biochar application rates, metabolite identification and classification were performed using the HMDB database. A total of 791 metabolites were annotated and grouped into 15 major categories (**Figure 5a**), The dominant metabolite classes included lipids and lipid-like molecules (32.49%), organoheterocyclic compounds (17.07%), organic acids and derivatives (13.78%), benzenoids (11.63%), organic oxygen compounds(9.23%), phenylpropanoids and polyketides (5.06%), along with nucleosides, nucleotides, and analogues (3.29%), respectively.

Metabolites in each biochar treatment were compared with CK, resulting in five metabolic sets (C5 vs. CK, C10 vs. CK, C20 vs. CK, C30 vs. CK, C40 vs. CK) based on the screening criteria of variable importance in projection (VIP) > 1 and *p* < 0.05. An upset diagram was employed to analyze the differences among these metabolic sets (**Figure 5b**). A total of 242, 263, 236, 265, and 241 differentially accumulated metabolites (DAMs) were identified in C5 vs. CK, C10 vs. CK, C20 vs. CK, C30 vs. CK, and C40 vs. CK, respectively. Meanwhile 35, 26, 8, 33, and 54 unique metabolites detected in each comparison. These results indicate that the greatest number of DAMs was observed in C30 vs. CK, whereas the highest number of unique DAMs was found in the C40 vs. CK treatment. **Figures 5c, d**, and S2c-S2e present volcano plots depicting metabolites that exhibit DAMs between biochar treatments and the CK. The number of DAMs upregulated was 127, 192, 142, 198, 105 while those downregulated were 115, 71, 94, 67, 136 in the C5 vs. CK, C10 vs. CK, C20 vs. CK, C30 vs. CK, and C40 vs. CK, respectively. These results indicate that biochar treatments significantly altered the metabolite profiles in cucumber rhizosphere soil.

#### 3.4.3 Differential metabolites KEGG pathway enrichment and key metabolic pathways

**Figure 6:**
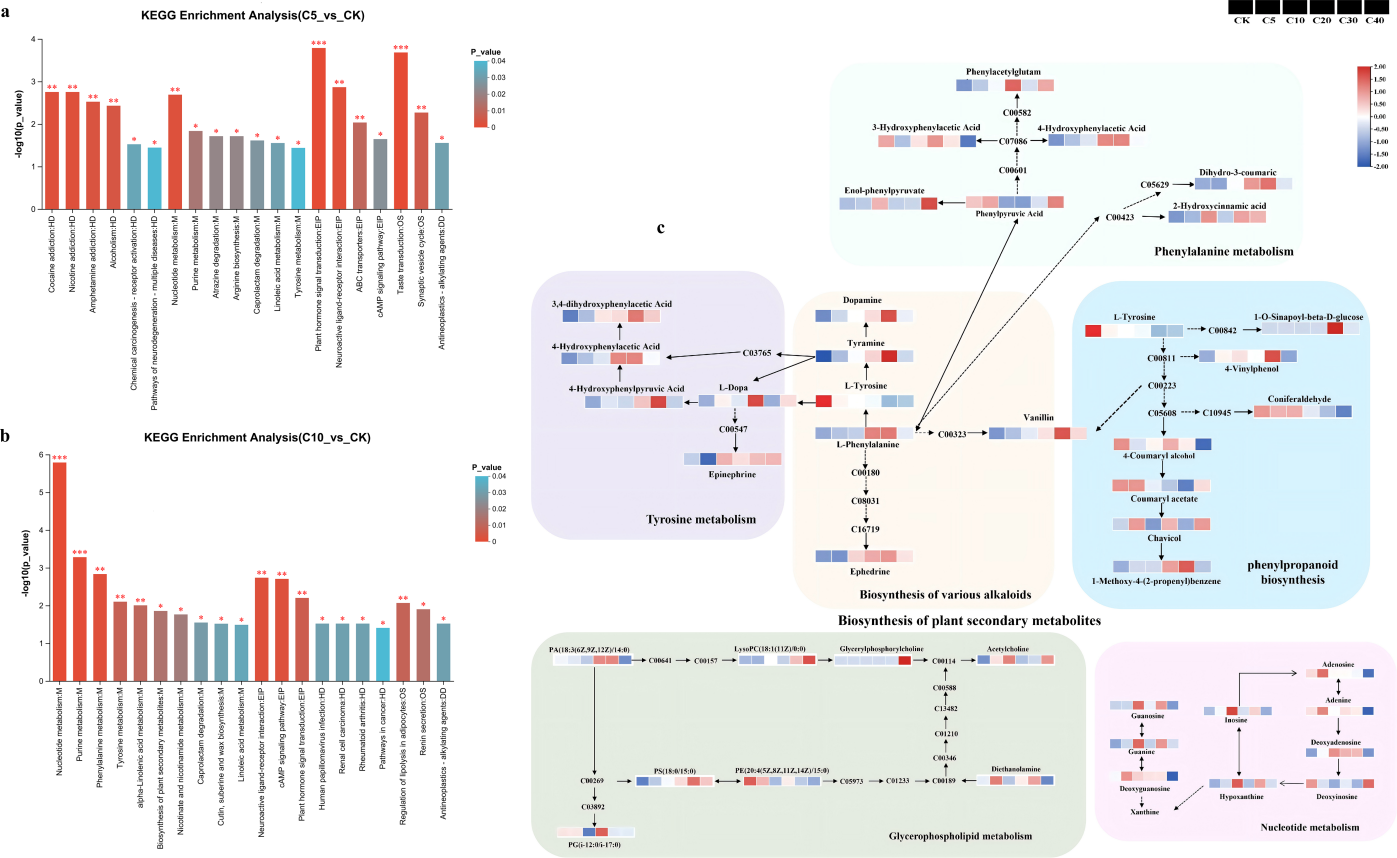
KEGG pathway enrichment analysis and important metabolic pathways for DAMs across biochar treatments and CK: **(a–c)** KEGG enrichment plots showing significantly enriched metabolic pathways for DAMsin **(a)** C5 vs. CK, **(b)** C10 vs. CK, and **(c)** screened key enrichment pathways.

**Figure 7:**
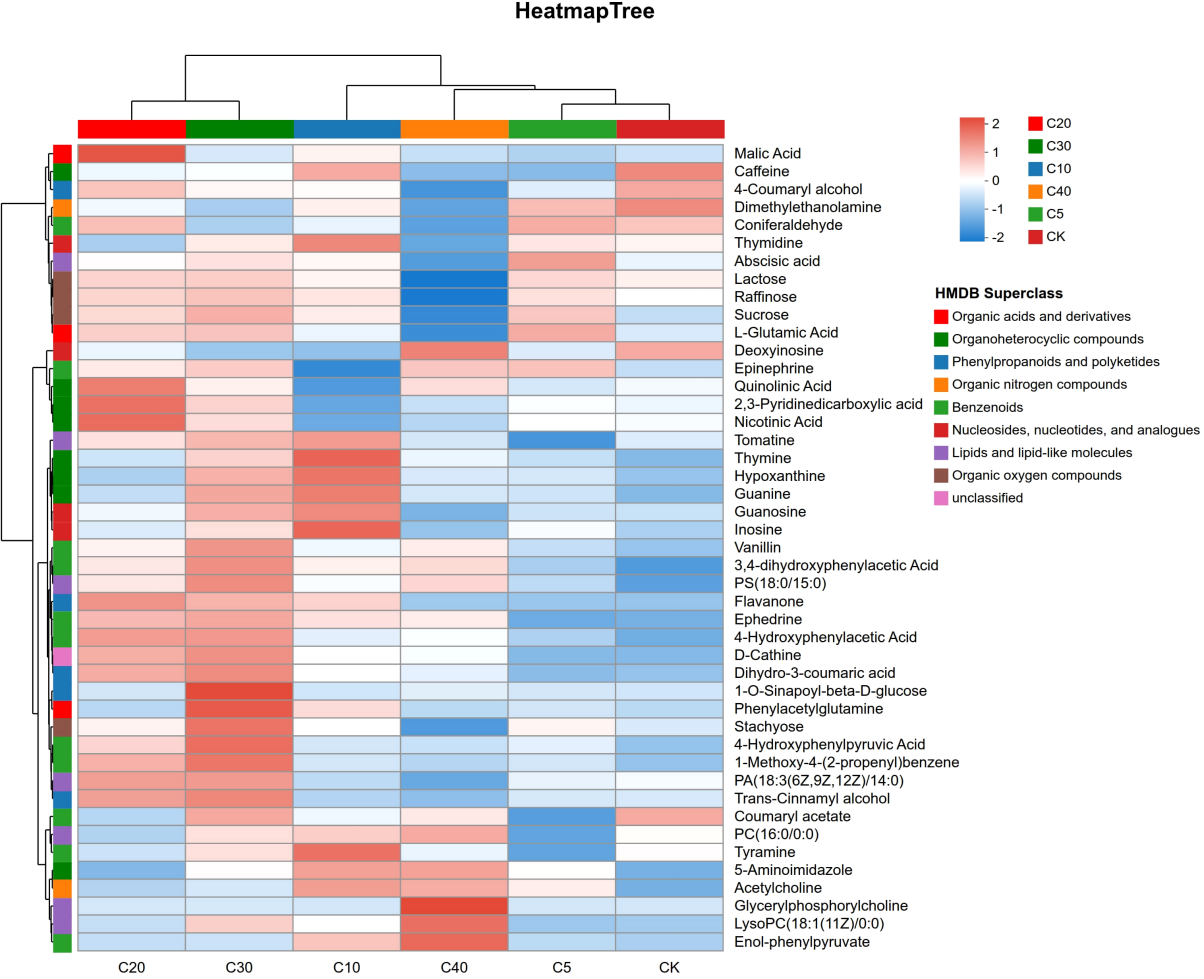
Cluster analysis of differential metabolites across biochar treatments and CK.

Metabolites exhibiting significant changes participate in multiple critical metabolic pathways. Significant differences in metabolite enrichment within each pathway were identified using Kyoto Encyclopedia of Genes and Genomes (KEGG) analysis, and the top 20 metabolic pathways differentiating biochar treatments from CK are illustrated in **Figures 6a, b**, and **Supplementary Figure S3c–e**. Specifically, 20, 20, 20, 13, and 5 significantly enriched metabolic pathways were detected in the comparisons of C5 vs. CK, C10 vs. CK, C20 vs. CK, C30 vs. CK, and C40 vs. CK, respectively. Differential metabolites were significantly enriched in plant hormone signal transduction, ABC transporters, and nucleotide metabolism in C5 vs. CK (*p* < 0.05, -log10(*p*) > 2; **Figure 6a**). In C10 vs. CK, significant enrichment was observed in nucleotide metabolism, purine metabolism, phenylalanine metabolism, tyrosine metabolism, cAMP signaling pathway, plant hormone signal transduction, and alpha-linolenic acid metabolism (*p* < 0.05, -log10(*p*) > 2; **Figure 6b**). For C20 vs. CK, differential metabolites were significantly enriched in the biosynthesis of plant secondary metabolites, tyrosine metabolism, plant hormone signal transduction, nicotinate and nicotinamide metabolism, and the biosynthesis of alkaloids derived from ornithine, lysine, and nicotinic acid (*p* < 0.05, -log10(*p*) > 2; **Supplementary Figure 3c**). In C30 vs. CK, significant enrichment was found in phenylalanine metabolism, nucleotide metabolism, phenylpropanoid biosynthesis, phenylalanine, tyrosine and tryptophan biosynthesis, biosynthesis of various alkaloids, and tyrosine metabolism (*p* < 0.05, -log10(*p*) > 2; **Supplementary Figure 3d**). Finally, in C40 vs. CK, differential metabolites were significantly enriched in glycerophospholipid metabolism, galactose metabolism, and plant hormone signal transduction (*p* < 0.05, -log10(*p*) > 2; **Supplementary Figure 3e**). Among the 17 major metabolic pathways identified, most were shared between two or more treatment groups. Based on these analyses, eight key metabolic pathways associated with continuous cropping stress resistance and yield improvement were identified, including nucleotide metabolism (mainly purine metabolism), biosynthesis of plant secondary metabolites (mainly tyrosine metabolism, phenylalanine metabolism, biosynthesis of various alkaloids, phenylpropanoid biosynthesis), glycerophospholipid metabolism, and galactose metabolism (**Figure 6c**). A total of 45 DAMs were enriched within these pathways and were categorized into nine groups (**Figure 7**). Cluster analysis of the treatment groups revealed that C5 and CK clustered together, which then grouped with C40 to form a larger cluster; this combined cluster subsequently grouped with C10. In contrast, C20 and C30 formed an independent cluster. These results indicated that biochar addition altered the relative abundance of metabolites, with metabolites exhibiting consistent responses at both low and high biochar application rates, while a different response pattern was observed at medium and high application rates. Compared with CK, the C30 treatment significantly upregulated most differential metabolites. The C10 treatment notably increased nucleotide metabolite content, whereas the C20 treatment significantly enhanced metabolites involved in the biosynthesis of plant secondary metabolites. The C40 treatment significantly upregulated lipid metabolites and significantly decreased galactose metabolites; conversely, treatments from C5 to C30 significantly increased galactose metabolite content.

### 3.5 Correlation of bacterial communities with soil physicochemical properties and soil metabolisms

The microbial community within the crop rhizosphere is impacted by various rhizosphere chemical properties mediated by root exudates (Li et al., 2020). The relationships between the rhizosphere bacterial community and soil properties, soil enzyme activities, yield, and metabolite profiles were further assessed using the Mantel test. TN, AP, SUC, URE, and yield were identified as the primary environmental factors impacting the bacterial community (*p* < 0.05).Additionally, most metabolites exhibited a significant positive correlation with the bacterial community (**Supplementary Figures 4a, b**).

**Figure 8:**
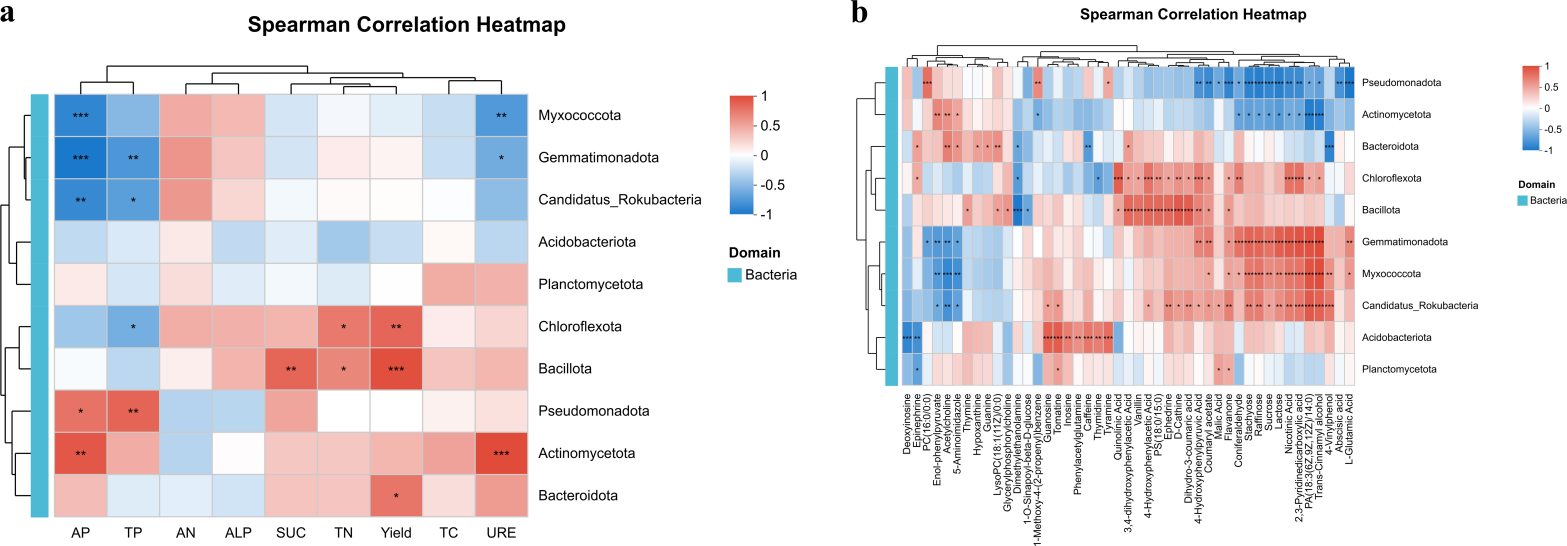
Spearman correlation between rhizosphere microbial community structure and soil environmental factors: **(a)** Correlation heatmap between key bacterial phyla and soil physicochemical properties, enzyme activities, and cucumber yield; **(b)** Correlation heatmap between bacterial phyla and DAMs. (Color intensity reflects the strength and direction of correlation (red = positive, blue = negative). Asterisks indicate significance levels: *p* < 0.05, *p* < 0.01, *p* < 0.001.).

Spearman correlation analysis was conducted to identify bacterial taxa that play pivotal roles in the variation of environmental factors and metabolites (**Figures 8a, b**). The results indicated that Myxococcota, Gemmatimonadetes, and Candidatus Rokubacteria exhibited positive correlations with AN and ALP, although these correlations were not statistically significant. These taxa were negatively correlated with other environmental factors and showed significant negative correlations with AP, TP, and URE. Furthermore, Myxococcota was significantly positively correlated with RS, whereas Actinobacteriota showed significant negative correlations with RS. In contrast, Proteobacteria and Actinobacteriota demonstrated significant positive correlations with AP, TP, and URE. Bacillota, Bacteroidetes, and Chloroflexi were significantly positively associated with yield. Notably, Chloroflexi and Bacillota were significantly positively correlated with TN, VC, and SP. Additionally, Bacillota showed a significant positive correlation with SUC, whereas Chloroflexi exhibited a significant negative correlation with TP (**Figure 8a**). In addition, Planctomycetota showed significant negative correlations with TS. Metabolites involved in galactose metabolism and the biosynthesis of plant secondary metabolites (involved in tyrosine metabolic synthesis, phenylalanine metabolic synthesis, phenylpropanoid metabolic synthesis, and the synthesis of various alkaloid metabolites) were significantly negatively correlated with Proteobacteria and Actinobacteriota but significantly positively correlated with Gemmatimonadota, Myxococcota, and Candidatus Rokubacteria. Furthermore, metabolites related to plant secondary metabolite biosynthesis were significantly positively correlated with Chloroflexi and Bacillota. Most metabolites associated with glycerophospholipid metabolism and nucleotide metabolism were significantly positively correlated with Bacteroidota, but showed significant negative correlations with Gemmatimonadota, Myxococcota, and Candidatus Rokubacteria. Metabolites associated with the nucleotide metabolism, amino acid metabolism (e.g., tyrosine and phenylalanine metabolism) and biosynthesis of various alkaloids were significantly positively correlated with Acidobacteria. (**Figure 8b**).

## 4 Discussion

### 4.1 Biochar improved cucumber yield and quality

Lu et al. (2022) demonstrated that biochar possesses substantial capabilities in regulating and restoring soil properties, as well as enhancing crop yield and quality. Consistent with these findings, this study revealed that varying biochar application rates significantly increased cucumber yield by 20% to 50%, with a positive correlation between crop productivity and biochar application rate and the most pronounced effect observed at application rates of 30 and 40 t ha⁻¹. This conclusion is consistent with findings from previous studies on rice, thin-skinned melons, and chili peppers (Wang et al., 2025; Xu et al., 2025; Zhang et al., 2025). Moreover, high biochar application rates have been shown to exert long-term legacy effects. Applications ranging from 20 to 100 t ha^-1^ can sustain soil ecosystem multifunctionality for up to 3 years (Rong et al., 2023). In particular, high-rate treatments (3%-4.5% w/w) significantly and durably enhanced soil nutrient availability and enzyme activities. Notably, in a 6-year field experiment, rice yields under the 3.0% (w/w) biochar treatment consistently outperformed all other treatments across the entire study duration (Jin et al., 2024). Cucumber fruit quality is determined not only by soil nutrients supply, but also by the plants’ efficiency in nutrient uptake and utilization. Increased yield exerted a significant positive regulatory effect on fruit vitamin C and soluble protein contents, a finding consistent with previous studies (Wu et al., 2022). Correlation analysis further confirmed a significant positive correlation between yield and both soluble protein (SP) and vitamin C (VC). Except for the C5 treatment, which significantly increased total sugar content, all other treatments reduced the contents of total sugar, soluble sugar and reducing sugar to varying degrees. This result aligns with the conclusion reported by Gao et al. (2025) (Gao et al., 2025), who reported that biochar enhanced sweet potato yield but significantly decreased the sucrose, glucose and fructose contents in storage roots. The underlying mechanism may be that biochar promotes plant uptake of nutrients such as nitrogen and phosphorus, thereby directing more photosynthetic carbon skeletons toward fruit cell division and expansion rather than being stored as soluble sugars and reducing sugars. This shift ultimately contributes to higher yield but reduced sugar accumulation. Correlation analysis also indicated a significant negative relationship between total sugar (TS) and yield.

### 4.2 Biochar improved soil physicochemical properties

In agricultural production, soil physicochemical properties and enzyme activities serve as critical indicators of soil fertility, quality, and health status (Wulanningtyas et al., 2021; Peng et al., 2023). In this study, medium and high biochar application rates (C20, C30, C40) all exhibited a positive promoting effect on soil nutrients and soil enzyme activities, and the C40 treatment produced a significant increase. Correlation analysis indicated that TN, URE, and SUC were significantly and positively correlated with yield. TN was also significantly positively correlated with SUC, while AP was significantly positively correlated with URE. TC was significantly positively correlated with SP. These results suggest that medium and high-rate biochar application can improve soil enzyme activities and nutrient contents, thereby facilitating nutrient uptake by plants and ultimately increasing cucumber yield and quality.

### 4.3 Biochar can modulate the rhizosphere microbiome

Soil microorganisms constitute essential components of the soil ecosystem. They are not only closely linked to variations in soil nutrients and enzyme activities but also exert a significant impact on crop yield and quality (Hernández-Lara et al., 2023; Song et al., 2023). In this study, the addition of biochar exerted a measurable impact on the diversity and richness of rhizosphere bacterial communities in soil, although these differences did not reach statistical significance. However, biochar application significantly altered the structure and composition of the rhizosphere soil bacterial communities. All biochar treatments notably enriched bacterial populations potentially active in the degradation of organic matter, soil nutrient cycling and carbon and nitrogen cycling, including members of the phyla Actinobacteria, Bacteroidetes, and Bacillota. Within Actinobacteria, the relative abundance of the dominant genera *Nocardioides*, *Streptomyces*, *Gaiella*, and *Ilumatobacter* increased significantly, with the C40 treatment yielding the highest values (**Supplementary Table 2**). Zhang et al. (2025) suggested that *Nocardioides* plays a critical role in organic matter transformation. *Streptomyces* contributes to enhanced nutrient uptake, crop growth promotion, and improved stress resistance (Niu et al., 2025). *Gaiella* is implicated in soil nitrogen cycling (Li et al., 2022) and the suppression of soil-borne pathogens (Wang et al., 2024; Wang et al., 2025). The carbon-rich nature of biochar likely underlies the observed increases in Bacteroidetes and Chloroflexi. Bacteroidetes and Chloroflexi were identified as key bacterial phyla contributing to yield and quality enhancement. High biochar application rates (C30) significantly increased the abundance of Bacillota, thereby accelerating organic carbon turnover and enhancing SUC activity. Conversely, the overall abundance of Proteobacteria decreased, potentially due to reductions in dominant genera such as *Hyphomicrobium*, *Bradyrhizobium*, and *Pseudolabrys*, which exhibited significant declining trends (**Supplementary Table 2**). These three genera jointly contribute to the coupling of soil carbon and nitrogen cycles by mediating key microbial processes such as nitrogen fixation, nitrification, and the degradation of organic carbon (Stone et al., 2021; Cui et al., 2024). In addition, biochar addition significantly reduced the abundance of the genus *Nitrospira*, this result aligns with previous studies (Li et al., 2020; Jiang et al., 2021; Cui et al., 2024) reporting that biochar inhibits nitrification by decreasing *Nitrospira* abundance. These shifts in microbial functional structure not only reshape the speciation and transformation efficiency of soil nitrogen, but also influence the nutrient supply potential and crop productivity of the soil ecosystem through the coupled dynamics of carbon and nitrogen cycling. The relative abundance of Acidobacteria decreased significantly following high biochar application (C40), likely because most Acidobacteria members are oligotrophic, improved soil nutrient conditions favored the proliferation of eutrophic bacterial phyla, including Actinobacteria and Bacteroidetes, resulting in a relative decline of Acidobacteria (Wang et al., 2024; Li et al., 2025). Changes in soil phosphorus levels exert a significant directional influence on the community dynamics of Gemmatimonadota, with high-phosphorus conditions markedly suppressing its abundance. This response is closely linked to the phosphorus retention capacity and ecological adaptability of Gemmatimonadetes (Liu et al., 2024), offering a microbial-level indicator for evaluating soil nutrient status. The synergistic optimization of multiple metabolic pathways involving nitrogen, carbon, and phosphorus likely accounts for the observed increases in TN and AP in the soil. This also reflects that biochar-mediated regulation of soil microbial communities exhibits distinct specificity toward microbial taxa. Collectively, these microbial community shifts provide a microbiological basis for the observed improvements in cucumber yield and quality.

### 4.4 Effect of biochar addition on soil metabolites

Soil metabolites are recognized as effective indicators for assessing changes in soil quality and function (Brown et al., 2024; Xu et al., 2024). Biochar application at varying rates induced distinct metabolic response patterns and pathway regulatory shifts in plants. At the low application rate (C5), metabolic responses were primarily associated with energy allocation and signal transduction. As the application rate increased from low-to-medium、medium-to-high levels (C30), the dominant responses progressively shifted toward enhanced biosynthesis of secondary metabolites. At the highest application rate (C40), lipid metabolism emerged as the principal metabolic pathway, while galactose metabolism was significantly downregulated. Nucleotide metabolism was enhanced under various biochar treatments, as evidenced by significant increases in the abundance of metabolites such as hypoxanthine, guanine, thymine, and inosine. Wang et al. (2023) suggested that the upregulation of the purine metabolism pathway has been shown to mitigate soil-related obstacles in continuous potato cropping systems. The biosynthesis of alkaloids and phenylpropanoids represents a central component of plant secondary metabolism (Li et al., 2025; Xia et al., 2025). Among the associated pathways, tyrosine metabolism and phenylalanine metabolism serve as critical metabolic hubs that link primary metabolism with the production of secondary metabolites. These pathways not only provide key precursors for the synthesis of alkaloids and phenylpropanoids but also function as core regulatory routes in the modulation of secondary metabolite biosynthesis. The tyrosine metabolism pathway accumulates various phenolic compounds, including 4-hydroxyphenylacetic acid, 3,4-dihydroxyphenylacetic acid, and 4-hydroxyphenylpyruvic acid. These compounds have been shown to scavenge reactive oxygen species or act synergistically with enzymatic reactions to effectively mitigate oxidative damage (Wang et al., 2023), a key defensive response of *plants-* to biotic and abiotic stress. The phenylalanine metabolism pathway is integral to regulating plant growth, enhancing stress resistance, and synthesizing secondary metabolites such as phenylpropanoids (Wang et al., 2023). Phenylpropanoid metabolism represents a classic metabolic pathway closely linked to plant induced resistance (Li et al., 2025). Within the biosynthesis pathway of plant secondary metabolites, multiple compounds were enriched, including organic acids (L-glutamic acid, malic acid) and phenylpropanoids and polyketides (flavanone, trans-cinnamyl alcohol, 1-O-sinapoyl-β-D-glucose). It has been reported that organic acids entering the soil positively affect plant ecological adaptability, thereby enhancing resistance to adverse environmental conditions and overall adaptability (Suh et al., 2021; Wang et al., 2023). Malic acid secreted by plant roots can recruit beneficial soil bacteria, modulate soil enzyme activity, and increase phosphorus availability, ultimately promoting plant growth (Xu et al., 2023). Phenylpropanoids and polyketides play crucial roles in plant defense responses, structural integrity maintenance, and survival fitness. (Wang et al., 2022; Cui et al., 2024). The degradation of phospholipids contributes to an increase in AP in soil (Yang et al., 2023). In this study, acetylcholine and phosphatidylcholine exhibited a significant positive correlation with AP (**Supplementary Figure 5**), further supporting the hypothesis that glycerophospholipid degradation products serve as phosphorus sources in the rhizosphere soil. Given that glycerophospholipids contain a nitrogenous head group (ethanolamine) as well as carbon, their decomposition represents a critical process in soil nutrient cycling, directly supplying essential elements required for the growth of *plants-* and microorganisms. This also accounts for the significant positive regulatory effects of glycerophospholipid and LysoPC(18:1(11Z)/0:0) on yield, URE activity, sucrose content, TN and TC, whereas dimethylethanolamine exhibits a significant negative regulatory effect (**Supplementary Figure 5**). Oligosaccharides function as both energy sources and structural components and can serve as carbon sources for plant growth and development (He, 2020). Among them, raffinose has been reported to facilitate root colonization by plant growth-promoting rhizobacteria (PGPRs) (Liu et al., 2017), whereas sucrose has been shown to inhibit the growth of the pathogenic fungus *Fusarium* spp. (Tian et al., 2022). This reduction may be attributed to the requirement of carbohydrate assimilation for glycerophospholipid synthesis (Xu et al., 2023), or to the increased nutrient demand of Proteobacteria and Actinobacteria during proliferation, leading to the consumption of these four sugars. This finding further explains the significant negative correlations between these oligosaccharides and the bacterial taxa Proteobacteria and Actinobacteria. In this study, Gemmatimonadetes, Myxococcota, Candidatus Rokubacteria, Chloroflexota, Bacillota, and Planctomycetota exhibited significant positive correlations with metabolites such as flavanone, trans-cinnamyl alcohol, 4-hydroxyphenylacetic acid, ephedrine, and PS (18:0/15:0). These results suggest that these bacterial taxa may play a major role in facilitating the secretion of stress-responsive metabolites.

Soil microorganisms play a pivotal role in soil metabolic activities, with their metabolic reactions directly reflecting microbial responses to the soil environment. Furthermore, variations in the composition and abundance of soil microorganisms partially regulate variations in soil metabolites, which in turn impact soil nutrient cycling and metabolic processes. Under biochar treatments, soil microorganisms significantly mediate changes in soil metabolism. Specifically, key bacterial taxa involved in organic matter degradation, nutrient cycling, and carbon-nitrogen-phosphorus cycling exhibit significant correlations with metabolites associated with carbon-nitrogen cycling and stress resistance. This result provides novel evidence supporting the hypothesis that soil metabolites may be secreted by microorganisms (Salcedo-Vite et al., 2019). Positive co-occurrence patterns suggest that these metabolites may be compounds secreted by microorganisms, whereas negative co-occurrence likely reflects the consumption or degradation of these metabolites by specific microbial taxa (Baker et al., 2024). Zhu et al. (2022) demonstrated that variations in amino acid metabolism, carbohydrate metabolism, pyrimidine metabolism, purine metabolism, and the biosynthesis of secondary metabolites are linked to enhanced plant resistance to abiotic stress. Given that metabolic pathways related to amino acids, carbohydrates, and lipids are all involved in carbon or nitrogen metabolism, the research results indicate that biochar treatment significantly enhances soil carbon and nitrogen metabolism and mitigates stress induced by continuous cropping obstacles.

## 5 Conclusion

This study demonstrates that soil physicochemical properties, soil microbial community, soil metabolites of the rhizosphere, cucumber yield and quality exhibited positive responses to biochar application in continuous cucumber cropping systems. Biochar treatments were significantly altered the rhizosphere microbial community structure by enriching core taxa such as Actinobacteria, Bacteroidetes, Bacillota, and Chloroflexi. Additionally, biochar significantly affect key metabolic pathways in plant roots, including lipid metabolism, the biosynthesis of various secondary metabolites, nucleotide metabolism, as well as the carbohydrate metabolism. The coordinated changes observed between the rhizosphere metabolome and microbiome under biochar treatments were intimately associated with soil carbon, nitrogen, phosphorus cycling and plant stress tolerance. Overall, biochar improves soil quality and enhances cucumber yield and quality under continuous cropping conditions by shaping rhizosphere bacterial communities and regulating root-associated metabolite profiles, with the medium-to-high application rates of biochar (30 and 40 t ha^-1^) exerting the most significant comprehensive positive effects, whereas, the medium application rates of biochar (20 t ha^-1^) better enhances fruit nutritional quality. These results provide valuable practical guidance for biochar application in mitigating continuous cropping obstacles, and offer a scientific basis for its application in advancing sustainable agricultural practices.

## Data Availability

The datasets presented in this study can be found in online repositories. The names of the repository/repositories and accession number(s) can be found in the article/**Supplementary Material**.
