# Biochar enhances cucumber production by modulating rhizosphere microbiota and soil metabolites under continuous cropping systems

**DOI:** 10.3389/fpls.2026.1899816

**Published:** 2026-06-12

**Authors:** Guoping Xue, Yun Hu, Haifeng Xue, Xueyu Wang, Hongmei Bai, Jinwei Du, Yaping Wang, Hongli Huo, Ming Li, Wei Jiang

**Affiliations:** 1 College of Horticulture and Plant Protection, Inner Mongolia Agricultural University, Hohhot, China; 2 Inner Mongolia Academy of Agricultural and Animal Husbandry Sciences, Hohhot, China; 3 Vocational and Technical College of Inner Mongolia Agricultural University, Baotou, China

**Keywords:** bacterial community, biochar, cucumber, metagenomic analysis, untargeted metabolomics

Affiliation 3 was omitted for author “Ming Li”. This affiliation has now been added for author “Ming Li”.

The original version of this article has been updated.

