# Supplementary material for: Biochar enhances cucumber production by modulating rhizosphere microbiota and soil metabolites under continuous cropping systems
Source: Front Plant Sci. 2026 May 21;17:1726191. doi: 10.3389/fpls.2026.1726191 (PMC13233690; doi:10.3389/fpls.2026.1726191)
Supplement: Supplementary file 1 [file DataSheet1.docx]

Supplementary Material

# Supplementary Figures and Tables

## Supplementary Figures


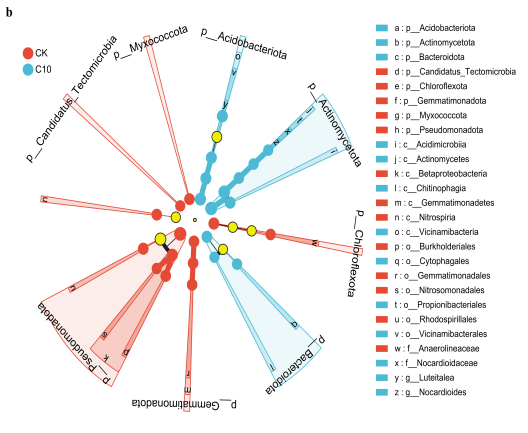

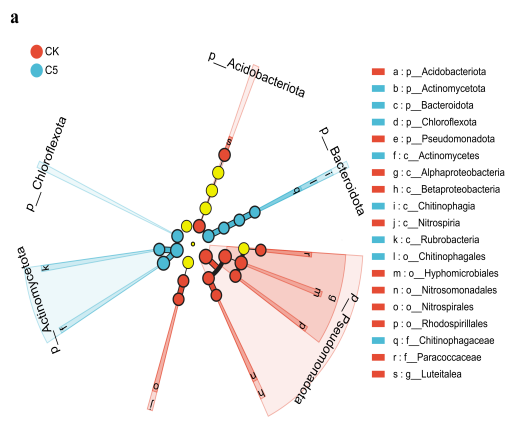

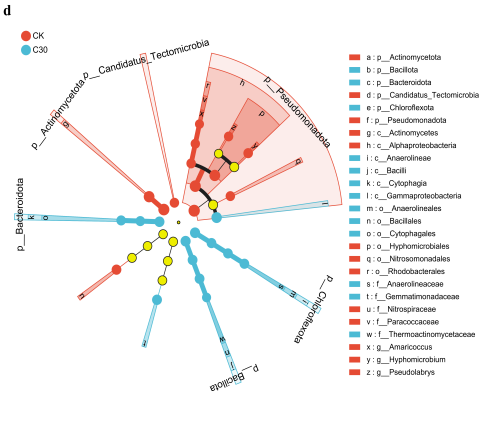

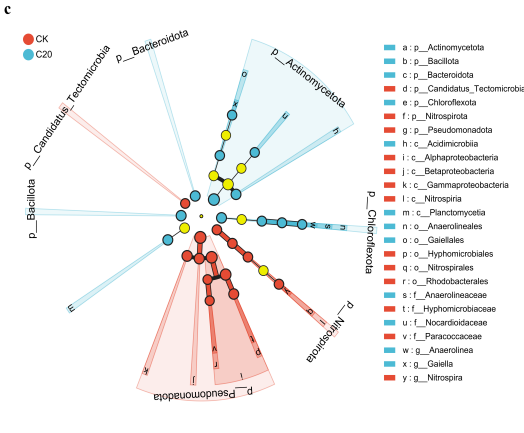


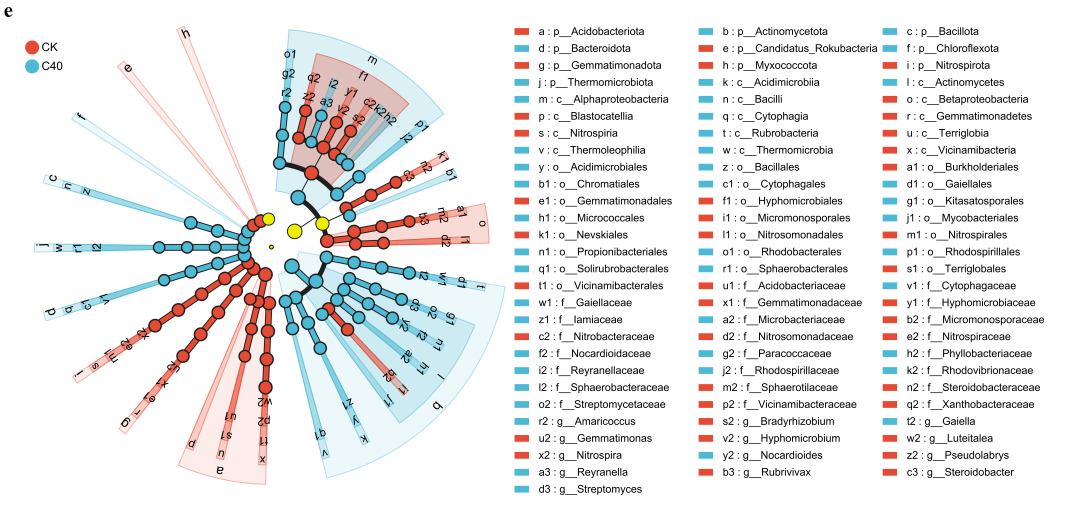


**Supplementary Figure 1.** Differential enrichment of rhizosphere bacterial communities under biochar treatments: (a-e) Linear Discriminant Analysis (LDA, score > 3) identifies significantly enriched taxa in C5vs. CK (a), C10 vs. CK (b), C20 vs. CK (c) , C30 vs. CK (d) and C40 vs. CK (e).

**Supplementary Figure 2.** Volcano plots of DAMs between treatments and CK: (c, d, e) Volcano plots showing the distribution of DAMs in C20 vs. CK (c), C30 vs. CK (d) and C40 vs. CK(e). (Note: Each dot represents a unique metabolite, with size indicating the VIP (Variable Importance in Projection) score. Red dots indicate significantly upregulated metabolites, green dots represent significantly downregulated metabolites, and blue dots denote metabolites with no significant change (VIP > 1 and p < 0.05).


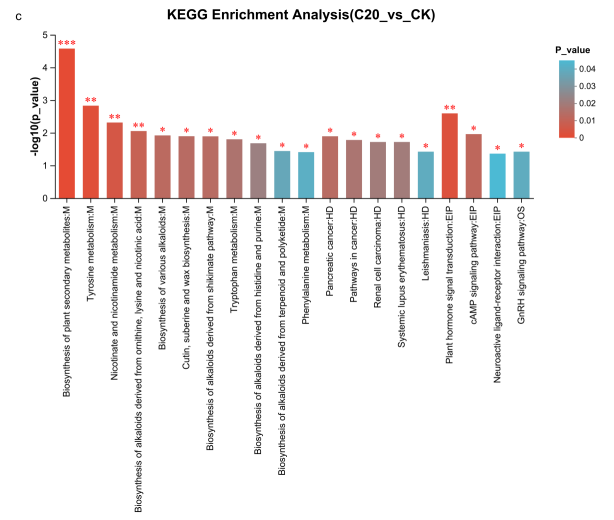

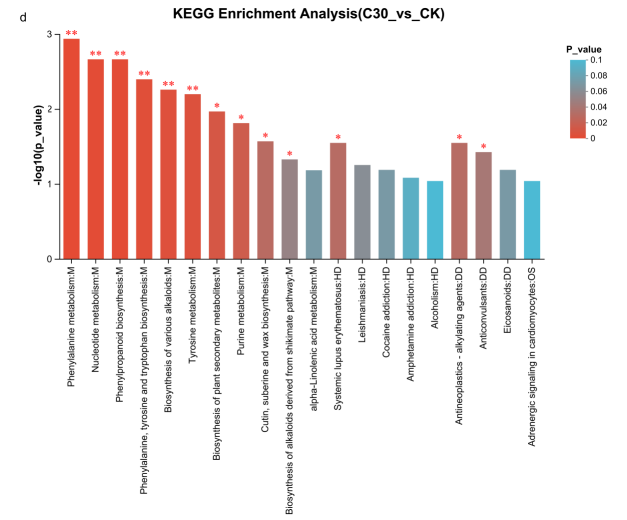

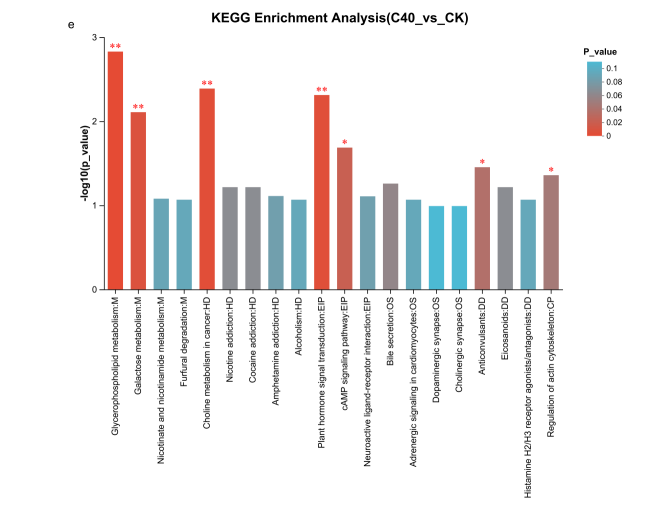

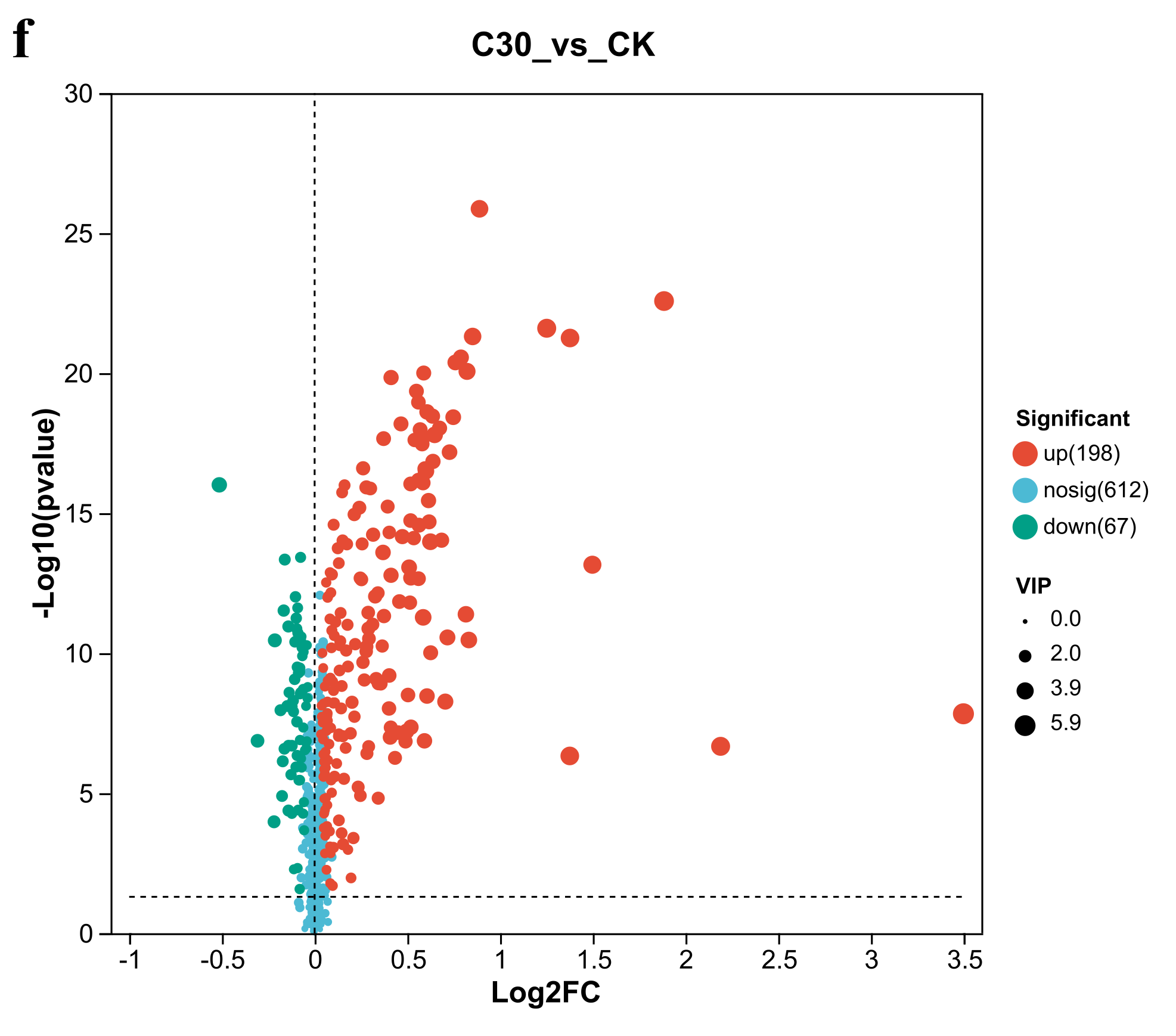

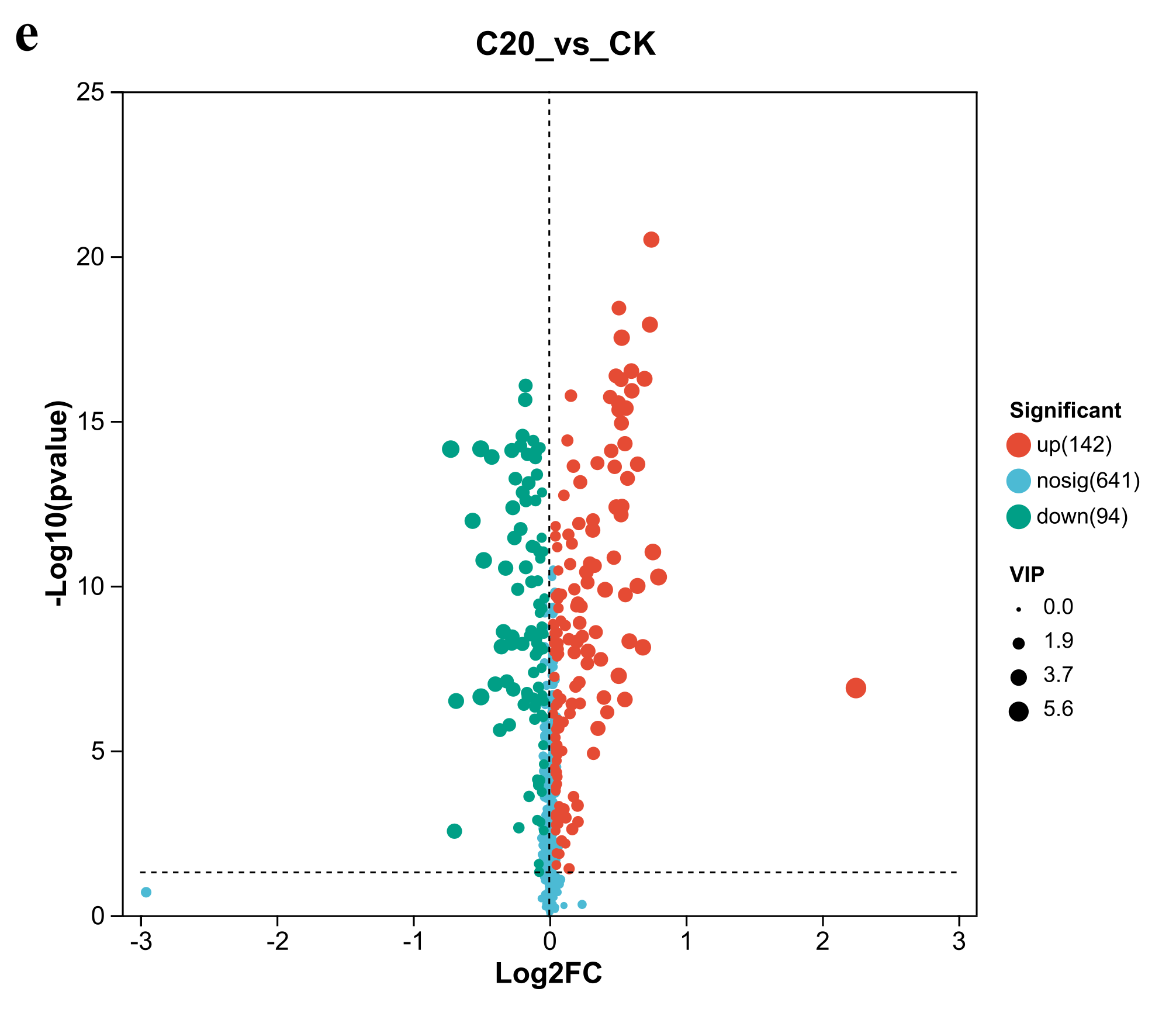

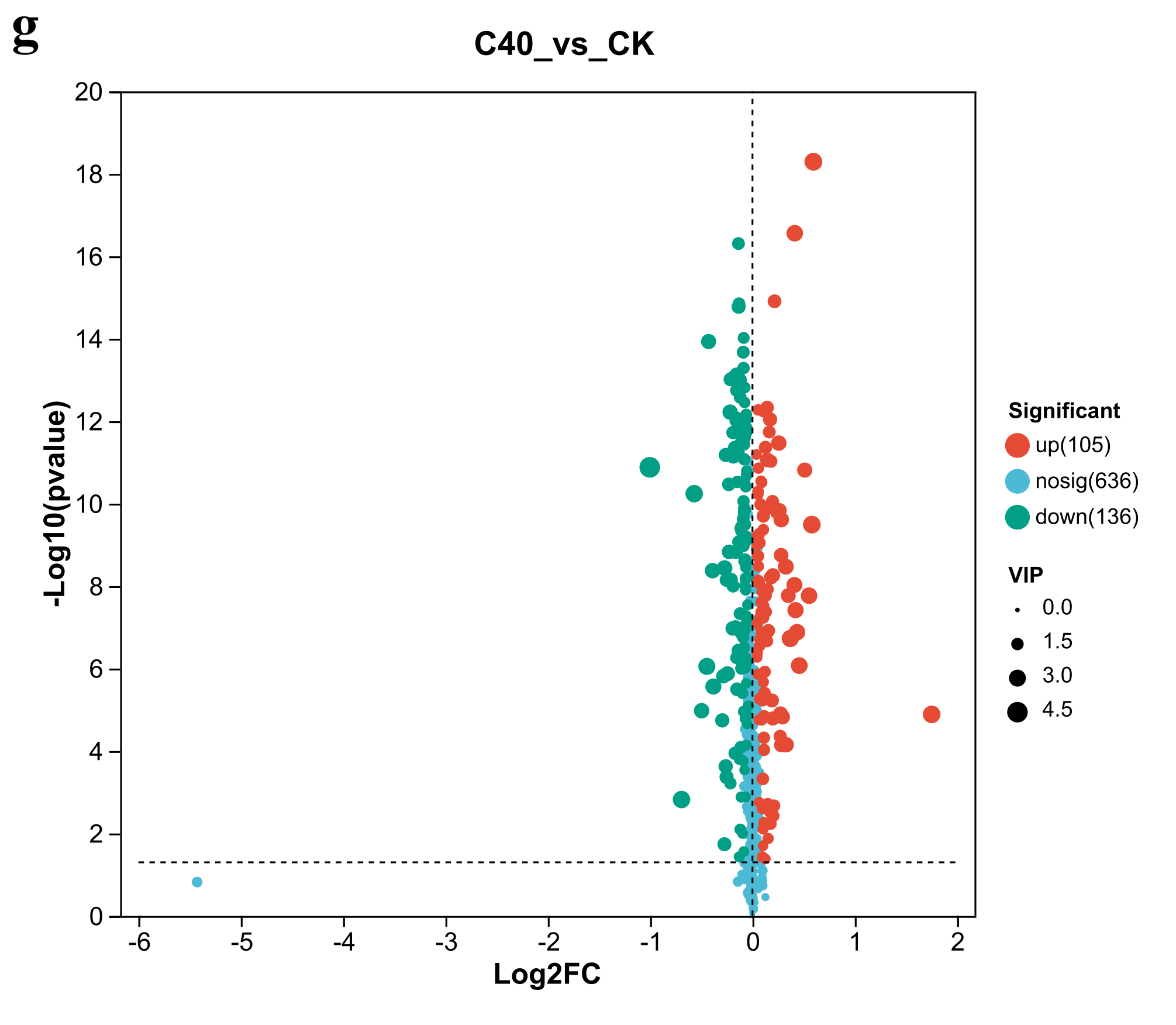


**Supplementary Figure 3.** KEGG pathway enrichment analysis of DAMs between biochar treatments and CK: (c, d, e) KEGG enrichment plots showing significantly enriched metabolic pathways for DAMs in C20 vs. CK (c), C30 vs. CK (d) and C40 vs. CK (e).Note: Asterisks indicate significance levels: *, ** and ***indicate *P* < 0.05, *P* < 0.01 and *P* < 0.001respectively.


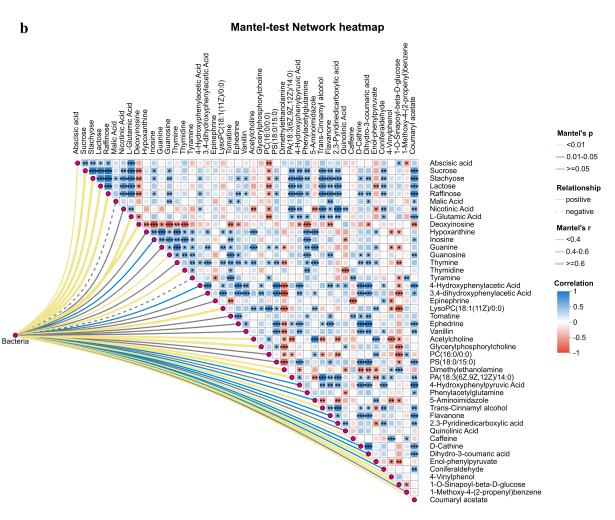

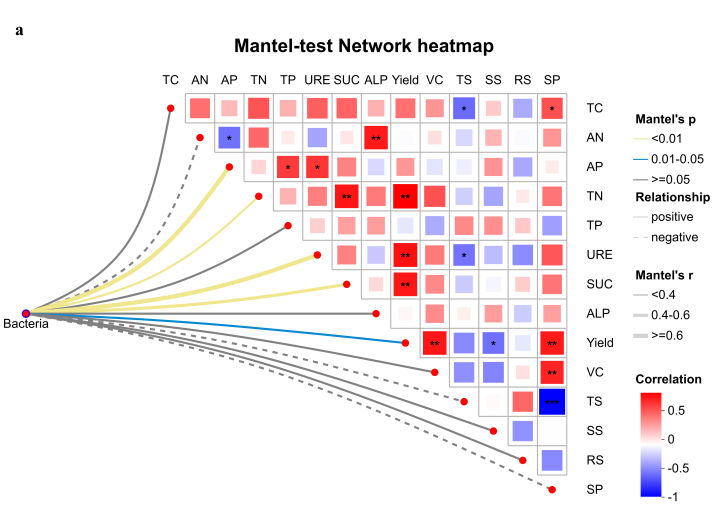


**Supplementary Figure 4.** Drivers of rhizosphere bacterial community structure revealed by mantel analyses: (a, b) Mantel test results indicating strength of association between bacterial community structure and soil properties (a), and between bacterial communities and metabolomic profiles (b).Note: Asterisks indicate significance levels: *, ** and ***indicate *P* < 0.05, *P* < 0.01 and *P* < 0.001respectively.





**Supplementary Figure 5.** Spearman correlation between DAMs and soil environmental factors: Correlation heatmap between DAMs and soil physicochemical properties, enzyme activities, and cucumber yield. (Note: Color intensity reflects the strength and direction of correlation (red = positive, blue = negative). Asterisks indicate significance levels: *, ** and ***indicate *P* < 0.05, *P* < 0.01 and *P* < 0.001respectively.)

## Supplementary Table

**Supplementary Table 1** Physicochemical properties of biochar used in experiment

| **Treatment** | **C%** | **N%** | **pH** | **EC**  **(μS･cm^-1^)** | **TN**  **(mg･g^-1^)** | **TP**  **(g･kg^-1^)** | **TK**  **(g･kg^-1^)** | **SOM**  **(g･kg^-1^)** | **AK**  **(g･kg^-1^)** | **AP**  **(g･kg^-1^)** | **AN**  **(mg･g^-1^)** |
| --- | --- | --- | --- | --- | --- | --- | --- | --- | --- | --- | --- |
| Biochar | 1.08 | 0.38 | 8.57 | 7460 | 0.726 | 1.29 | 2.08 | 358.85 | 17.16 | 0.37 | 29.36 |

**Supplementary Table 2** Significance analysis of differences at the genus level

| Pathway Description | C5vsCK | C10vsCK | C20vsCK | C30vsCK | C40vsCK |
| --- | --- | --- | --- | --- | --- |
| g__Luteitalea | ↓ | ↑** | ↓ | ↑ | ↓*** |
| g__unclassified_p__Candidatus_Rokubacteria | ↓** | ↓* | ↑** | ↑* | ↓*** |
| g__Nitrospira | ↓*** | ↓*** | ↓*** | ↓*** | ↓*** |
| g__Hyphomicrobium | ↓ | ↑ | ↓ | ↓*** | ↓*** |
| g__Gaiella | ↑*** | ↑ | ↑*** | ↓ | ↑*** |
| g__Gemmatimonas | ↑ | ↓*** | ↑ | ↑ | ↓*** |
| g__Anaerolinea | ↑*** | ↓*** | ↑*** | ↑*** | ↑** |
| g__Methyloceanibacter | ↑ | ↓ | ↓ | ↓ | ↑*** |
| g__Nocardioides | ↑*** | ↑*** | ↑*** | ↑*** | ↑*** |
| g__Bradyrhizobium | ↓*** | ↑ | ↓*** | ↓*** | ↓*** |
| g__Ilumatobacter | ↑*** | ↑** | ↑** | ↓ | ↑*** |
| g__Mesorhizobium | ↓*** | ↓ | ↓*** | ↓*** | ↑*** |
| g__Pseudolabrys | ↓*** | ↓*** | ↓*** | ↓*** | ↓*** |
| g__Steroidobacter | ↓ | ↑ | ↑ | ↑ | ↓*** |
| g__Streptomyces | ↑ | ↑** | ↑ | ↑ | ↑*** |
